# Visually induced postural reactivity is velocity-dependent at low temporal frequencies and frequency-dependent at high temporal frequencies

**DOI:** 10.1007/s00221-013-3592-3

**Published:** 2013-06-04

**Authors:** J.-M. Hanssens, R. Allard, G. Giraudet, J. Faubert

**Affiliations:** 1 Laboratoire de psychophysique et de perception visuelle, École d’optométrie, Université de Montréal, CP 6128, succ. Centre-ville, Montreal, QC H3C 3J7 Canada; 2 Départment de physiologie, Faculté de médecine, Université de Montréal, Montreal, Canada; 3 Essilor Canada, Montreal, Canada

**Keywords:** Velocity, Frequency, Postural response, Postural saturation

## Abstract

Visual stimulation alone is sufficient to produce visually induced postural reactivity (VIPR). While some studies have shown that VIPR increases with the velocity of a moving visual stimulus, others have shown that it decreases with the temporal frequency of an oscillating visual stimulus. These results seem contradictory given that these two variables co-vary in the same direction. The purpose of this study is to determine whether the VIPR can be different depending on the frequency range being considered. Twelve subjects were placed standing up in a virtual reality environment that simulated a black and white checkerboard at floor level. This checkerboard oscillated at seven frequencies (0.03–2.0 Hz) and three amplitudes (2, 4, and 8°), corresponding to nine velocities (0.125–32°/s). The virtual floor oscillated from left to right (mediolateral) or from front to back (anteroposterior). We calculated the subjects’ mean velocity (Ω) based on data from electromagnetic sensors positioned on the head and lower back. Our experiment shows that for temporal frequencies below 0.12 Hz, VIPR is visually dependent and increases with stimulus velocity. When stimulus velocity becomes too high, the body becomes incapable of following, and the VIPR saturates between 0.12 and 0.25 Hz. In this frequency range, maximal postural oscillation seems to depend on biomechanical constraints imposed by the positioning of the feet. For frequencies above 0.5 Hz, the body can no longer maintain the same oscillation state. This saturation may be linked to proprioceptive feedback mechanisms in the postural system.

## Introduction

Postural control relies on mechanisms that involve several different sensory systems. Vision plays a particularly important role in the upright stance, in which the vestibular and somatosensory systems are less solicited (Mahboobin et al. 2005; Oie et al. 2002). Numerous studies have tried to determine the properties of the postural response as a function of the temporal frequency and velocity of the visual stimulus. However, the respective contributions of these two parameters have not yet been clearly established. Although velocity is a function of amplitude and frequency, the visual stimulus velocity can vary with the amplitude with no changes in frequency and vice versa. In this study, we placed subjects in a virtual reality environment and exposed them to a variety of visual stimulus conditions in order to determine whether the postural response is induced primarily by the frequency of the stimulus or, conversely, by its velocity.

When a subject in a quiet standing position is confronted with a noteworthy change in their visual environment, a compensatory postural response is registered, enabling the subject to adjust to the new properties of the stimulus (Dokka et al. 2009). This is called visually induced postural reactivity (VIPR). In the literature, postural response has been defined as the amount of linear or angular body displacement (Berencsi et al. 2005; Dokka et al. 2009; Jeka et al. 2004; Lestienne et al. 1977; Sparto et al. 2006) or body velocity (Cornilleau-Peres et al. 2005; Jeka et al. 2004; Nougier et al. 1998) in regard to visual stimulation characteristics. Several authors also refer to the gain of the body amplitude as a function of the visual stimulation amplitude. Numerous studies have tried to determine what visual characteristics are most likely to generate such postural readjustment. Literature shows that postural behavior is influenced by spatial characteristics of the visual environment (Barela et al. 2011; Kunkel et al. 1998; Paulus et al. 1989). But what happens if the scene is moving? Some studies have focused on VIPR as a function of temporal frequency. They have found that the body excursion is maximal at low stimulus frequencies of about 0.25 Hz (Sparto et al. 2006; Peterka 2002) and then decreases with increasing frequency (Musolino et al. 2006; Sparto et al. 2006; van Asten et al. 1988a, b). Other studies have examined postural response as a function of stimulus velocity and found that VIPR increases with velocity (Dokka et al. 2009; Lestienne et al. 1977; Masson et al. 1995). These various results suggest that VIPR may be dependent on both stimulus frequency and velocity, increasing with velocity and decreasing with frequency. This behavior would seem contradictory given that these two variables co-vary in the same direction. However, this contradiction can be explained by the range of frequencies being considered in these different studies. It appears that those experiments that obtained a frequency-dependent postural response generally used relatively high frequencies in the range of 0.1–1 Hz (Musolino et al. 2006; Sparto et al. 2006; van Asten et al. 1988a, b), while those studies that found a velocity-dependent response examined lower frequencies in the range of 0.05–0.55 Hz (Dokka et al. 2009; Lestienne et al. 1977; Masson et al. 1995). Based on the findings of this previous research, we hypothesize that VIPR shows different degrees of sensitivity to the characteristics of the dynamic visual scene depending on the temporal frequency of the movement. In this study, we examine a wide range of temporal frequencies in order to cover the majority of frequencies and velocities reported in the literature.

A number of studies have compared the postural response in the mediolateral (ML) and anteroposterior (AP) directions, but most of these studies used static visual stimuli. It appears that postural oscillation is generally greater in AP than in ML (Anand et al. 2003; Winter et al. 1990; Paulus et al. 1989). Perrin et al. (1997) have even demonstrated that having the eyes open versus closed has very little effect on postural maintenance in ML, while postural stabilization in AP is significantly enhanced when the eyes are open. More recently, a study showed that changing the characteristics of the visual field alters the postural response in AP but has no effect in ML (Berencsi et al. 2005). These results suggest that postural stabilization in AP depends more on the spatial characteristics of the visual stimulus than is the case in ML. But what are the effects of the temporal characteristics of the stimulus on postural response? Based on these results, one could imagine that a dynamic visual stimulus would induce a greater postural response in AP than in ML. In two separate experiments, van Asten et al. (1988a, b) used a black and white windmill or tunnel pattern subjected to different types of movement: AP translation (van Asten et al. 1988b) and ML rotation around a longitudinal axis (van Asten et al. 1988a). Comparing the results of these studies shows that the maximal postural response induced by the stimulus movement was greater in ML than in AP. This suggests that the visual control of postural response in AP and ML varies differently depending on the spatial and temporal characteristics of the stimulus. However, it is difficult to draw conclusions about the postural response in a dynamic situation because while the stimuli were visually similar in these studies and the nature of the movement was not (i.e., ML oscillation vs. AP translation). In our study, we control this aspect by using a stimulus whose movement is identical in AP and ML: A virtual floor that sways from front to back (AP) or from left to right (ML). A greater maximal response in AP than in ML in our study would suggest that VIPR is similar in dynamic and static conditions. Conversely, a maximal response that is greater in ML than in AP would support the results of van Asten et al. (1988a, b).

## Methods

### Subjects

Four female and eight male volunteers took part in this study. The mean age was 24.25 ± 1.54 years (mean ± SD). At recruitment, none of the subjects reported any known visual and postural problem. All participants had a complete visual exam at the Clinique Universitaire de la vision, Université de Montréal. Only subjects with a monocular visual acuity of 20/20 with both eyes and a stereoscopic acuity better than 40 s of arc without glasses were accepted. We also did not enroll individuals that required wearing visual correction such as glasses or contact lens; this was to avoid any possibility of optical distortion altering the visual quality of stimuli. All of the participants gave their informed consent before beginning the study. The University of Montreal’s Health Research Ethics Board [Comité d’éthique de la recherche en santé (CERES) de l’Université de Montréal] approved the study.

### Apparatus

**Fig. 1:**
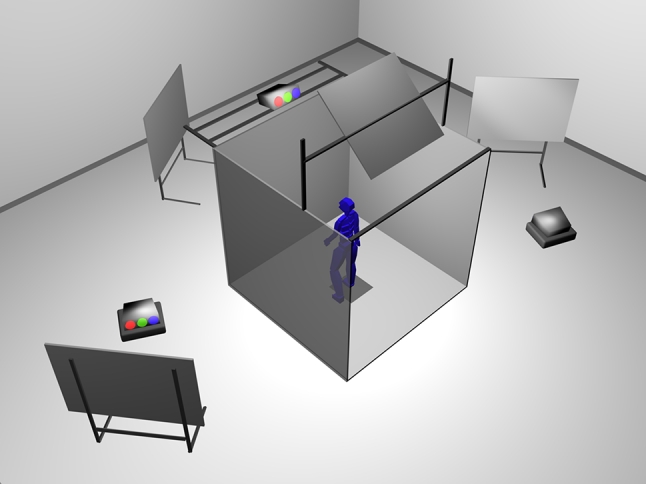
Setup of the full immersive virtual environment (FIVE)

The subjects were placed in a fully immersive virtual environment (FIVE; Fig. 1). The FIVE is a semi-open room that measures 8 × 8 × 8 feet and is enclosed by three walls (a front and two sides) and a floor. The visual stimuli were displayed on these four surfaces by Marquee 8500 Ultra projectors (Christie Digital Systems) with a resolution of 1,280 × 1,024 pixels. Three-dimensional vision was stimulated by the wearing of Crystal Eyes wireless shutter glasses (StereoGraphics). The display frequency of the 96-Hz screens (48 Hz per eye) was synchronized with that of the glasses; this isolated the images perceived by each eye, enabling the subject to see in three dimensions. SGI Onyx 3200 servers equipped with Infinite Reality 2 cards generated the virtual immersion in real time.

A virtual environment has been preferred over real visual stimulation because it allows a more flexible manipulation of the visual environment. Also, virtual reality systems make it possible to isolate the visual stimulation from proprioceptive, vestibular, or auditory stimulations. In such setups, proprioceptive and vestibular systems are not directly stimulated by the visual stimulus. In our experimental context, the visual stimulus was inducing postural response, whereas proprioceptive and vestibular systems were used to maintain the upright posture.

An effective way to induce virtual reality is to generate the visual stimulus in real time based on movements of the participants’ head. We placed an electromagnetic tracking system (Flock of Birds, Ascension Technology) in the 3D glasses for this purpose and another sensor on the lower back for measuring the subject’s postural stability. Data were converted from analog to digital at a sampling frequency of 64 Hz. A reported disadvantage using a virtual reality system is the lag between the subject’s movements and the incorporation of this information into the visual display (Akiduki et al. 2003). This lag here would be due to the Flock of Birds magnetic system update rate (about 80 Hz or 12.5 ms) and SGI servers frame rate (48 Hz or 21 ms per eye). The total lag was about 33.5 ms. Such a short delay was quite imperceptible by the participants. Furthermore, the average head movement in the present study was 0.46° ± 0.59 (1.41 cm ± 1.79). Such a small head movement induced a negligible adjustment of the virtual image with no noticeable distortion of the virtual floor.

### Visual stimuli

**Fig. 2:**
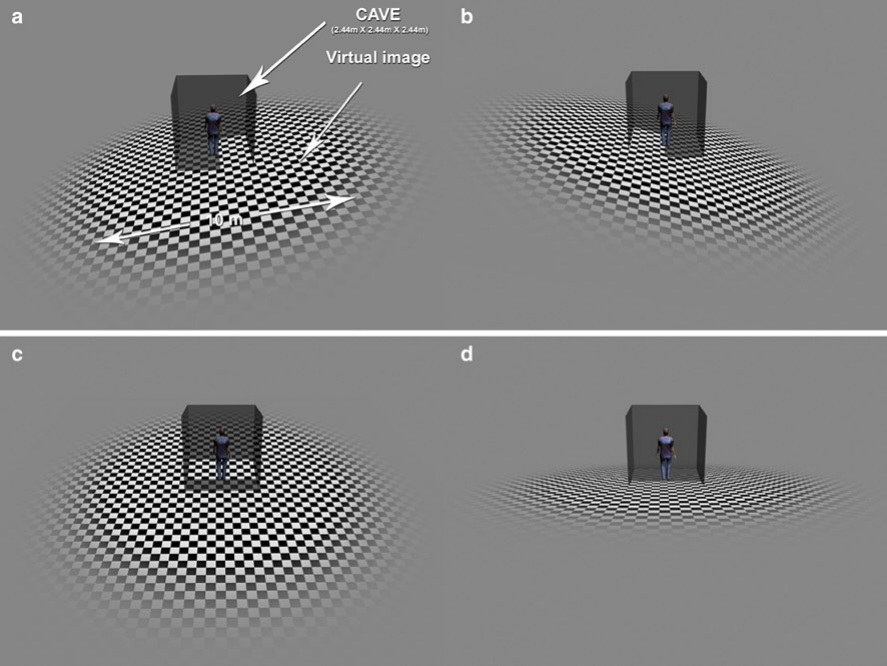
The visual stimulus used in the experiments. The subject was placed at the center of the FIVE. The subject perceived the checkerboard virtual floor at their feet, which oscillated in a *mediolateral direction* (**a**, **b**) or in an *anteroposterior direction* (**c**, **d**). The FIVE is shown in order to illustrate the experimental context; note that the subject could not perceive the screens, once the stimulus was activated

The floor is an important visual reference for maintaining postural control for a human in the upright position (Baumberger et al. 2004). Furthermore, a moving virtual floor is known to induce a significant postural response (Faubert and Allard 2004). In this experiment, the visual stimulus consisted of a black and white checkerboard virtual floor positioned at the level of the real floor (Fig. 2). As the checkerboard stimulus was wider than the floor screen, the visual stimulation was also simultaneously displayed on the three other screens. The virtual squares measured 25 cm on each side. The maximum brightness was 47 cd/m^2^ for the white squares, and 0.52 cd/m^2^ for the black squares (Michelson contrast = 98 %). The contrast of the virtual floor was maximal over a diameter of 10 meters and then gradually blurred to a gray color. It was positioned such that the subject was at its center.

To properly compare VIPR in the AP and ML directions, the same characteristics and equations of movement have been used for these two directions. One cannot use, for example, an oscillatory movement in one direction and a translation movement in the other. We therefore used an oscillatory movement for both the AP and ML conditions: swaying from side to side in the ML direction, and from front to back in the AP direction. The visual movement perceived by the subjects was similar to a ship pitching and rolling centered on the subject’ feet (Fig. 2). In order to manipulate temporal frequency and velocity simultaneously and avoid having to run two separate experiments (cf. van Asten et al. 1988a, b), we had the virtual floor oscillate in a sinusoidal movement characterized by the amplitude, frequency, or a combination of these two, velocity. We chose an oscillatory rather than a translation movement because the angular velocity of the virtual floor was similar to the oscillation velocity of the subjects. The sinusoidal function of the movement was defined by its amplitude *θ*_max_ (°), temporal frequency *f* (Hz), initial phase *p* (cycle), and time *t* (s).

$$\theta (t) = \frac{{\theta_{ \rm{max} } {\text{sin(}}2{{\uppi}}(ft + p) )}}{2}$$

The initial phase (*p*) was random. We chose peak-to-peak amplitudes (*θ*_max_ = 2, 4, 8°) and temporal frequencies (*f* = 0.03125, 0.0625, 0.125, 0.25, 0.5, 1, and 2 Hz) of the oscillation to be similar to the literature (Sparto et al. 2006; Loughlin and Redfern 2001). These frequency–amplitude combinations enabled us to obtain nine stimulus mean velocities of 0.125–32°/s. Only nine velocities were obtained instead of twenty-one because several frequency by amplitude combinations resulted in the same stimulus velocity. This approach also enabled us to vary only the temporal frequency or velocity (by controlling the amplitude). The stimulus was perceived through stereoscopic glasses with a large visual field (approximately 130° horizontal and 85° vertical). To create the virtual reality effect, the display was re-calculated in real time and as a function of the subject’s position.

### Experimental procedure

On the first day of the experiment, we measured the subject’s visual acuity and stereoscopic vision at a distance of 1.25 m to make sure they had good perception of the stimuli on the central screen. The subjects stood at the center of the FIVE (at 1.25 m from the central screen) with their feet together, wearing no shoes, and with arms crossed over their chest (Kawakita et al. 2000; Mahboobin et al. 2005). The instructions were to focus on a red fixation point situated at a virtual distance of 6 meters in front of them at floor level and to not make any voluntary movements during the experiment. The experiment was divided into two sessions spaced 24 h apart in order to avoid causing fatigue. Each of these two sessions covered one stimulus direction, with the order being chosen randomly. Each session comprised a total of 22 visual conditions: three amplitudes, seven frequencies, plus one control condition in which the checkerboard remained static. The order of these conditions was determined at random. Each condition lasted 68 s, followed by a 60-s pause. The sensor system recorded the postural response during presentation of the visual stimulus.

### Data analysis

The subject’s position was recorded in real time by the sensor on the head and lower back. However, given that our data showed the same trend for both sensors, we used the data for the head sensor only for analysis given that it was closer to the emitter consequently with lower noise levels. For all experimental conditions, we generated the power spectral density in MATLAB using a fast Fourier transformation (FFT). This analysis revealed a unidirectional spectrum for posture as a function of time for both the mediolateral axis, when the floor swayed from side to side, and for the anteroposterior axis, when the floor swayed front to back. The amplitude of the postural response (peak-to-peak) at the temporal frequency of the stimulus was extracted to yield the frequency-specific body sway amplitude (Sparto et al. 2006).

Linear displacements of the head were converted to angular displacements in order to control for the effect of body size (Greffou et al. 2008, 2011) and to compare body sway amplitude to the angular amplitude of the visual stimuli. Postural response gain is the ratio of frequency-specific body sway amplitude to the angular amplitudes of the visual stimulus. A subject whose postural response is perfectly aligned with the amplitude of the visual stimulus would have a gain of 1, while a postural response of lower amplitude than the visual stimulus would yield a gain of less than 1. In order to eliminate transitory postural responses that occur during the first few seconds just after the stimulus appears, we limited the analysis to the last 64 s of stimulus presentation (out of the total 68 s). We decided on the 64-s duration because it covers two complete cycles for the slowest frequency (0.03125 Hz), for which the period was 32 s.

The saturation of the postural response to a visual stimulus is shown in the subject’s velocity (Mergner et al. 2005). We used body sway amplitude (peak-to-peak) to calculate the mean velocity Ω of the subject’s postural response for each amplitude, frequency, and direction condition.

$$\Upomega (^\circ /{\text{s}}) = \frac{{2 \times {\text{body}}\; {\text{sway}}\;{\text{amplitude}}(^\circ )}}{{{\text{period}}({\text{s}})}}$$

### Statistical analysis

We used multi-factor repeated measures ANOVAs to test for the effects of the stimulus frequency, velocity, and direction on the subject mean velocity. A post hoc Bonferroni correction was made to adjust the conditions in multiple comparisons. We used paired samples *t* tests to analyze saturation of the postural response. The significance threshold was set at 0.05. The statistical analyses were carried out using SPSS Statistics 19.0 (SPSS Inc., LEAD Technologies Inc.).

## Results

### Gain

**Fig. 3:**
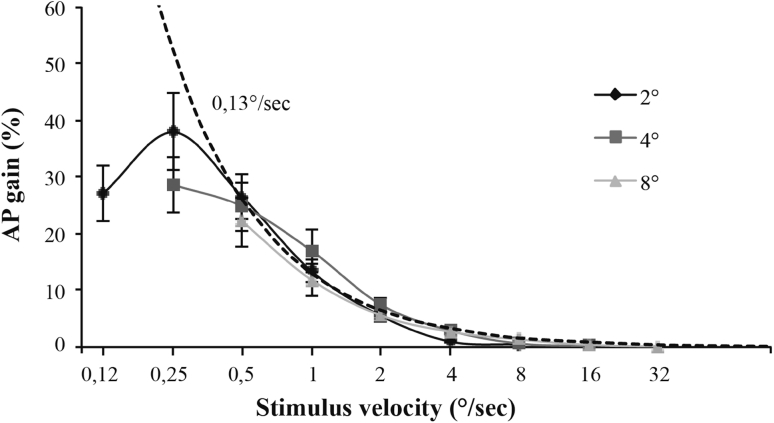
Mean postural response gain as a function of stimulus velocity in *anteroposterior direction* when the virtual floor sways from *front* to *back*. *Dashed curve* shows a theoretical gain that would occur if the body peak velocity always remained at 0.13°/s. The *error bars* represent standard errors

We will first present the results for the AP condition in order to compare our results to those of Mergner et al. (2005). Figure 3 shows postural response gain as a function of the velocity of the visual stimulus for the AP condition. Each curve represents one of the three stimulus amplitudes. The results show that postural response gain decreased when increasing stimulus velocity irrespective of stimulus amplitudes [*F*_(8, 88)_ = 29.0,67; *p* = 0.012]. As reported by Mergner et al. (2005), this decrease seems to be a hyperbolic decay. Calculating the AP body peak velocity for each point of the hyperbolic decay showed that body velocity always remains close to 0.13°/s. In order to better illustrate this point we added, to the Fig. 3, a theoretical gain curve (dashed) that would occur if the body peak velocity always remained at 0.13°/s regardless of the stimulus amplitude. This dashed curve overlaps relatively well the experimental data, suggesting that VIPR tends to saturate when postural response reaches a velocity of about 0.13°/s.

### Mean subject velocity

**Fig. 4:**
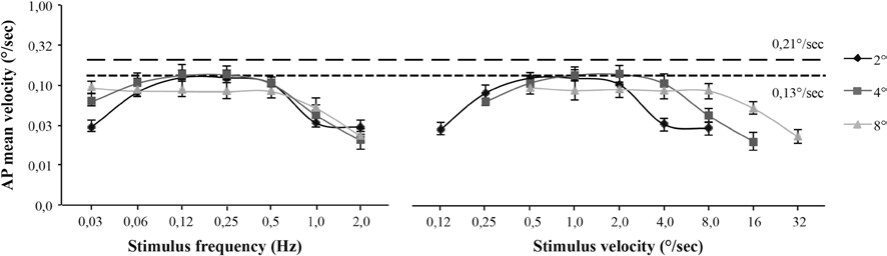
Anteroposterior mean velocity of subjects as a function of stimulus temporal frequency (*left*) and velocity (*right*). Results are presented for the three stimulus amplitudes. *Two straight lines* showing theoretical saturation, at 0.13 (*small dashes*) and 0.21°/s (*large dashes*), show the saturation points for AP and ML, respectively. The *error bars* represent standard errors

**Fig. 5:**
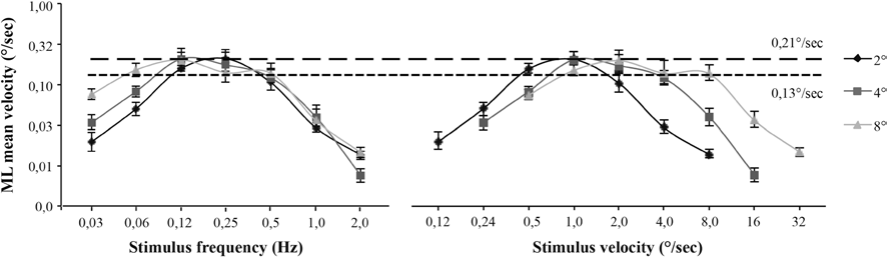
Mediolateral mean velocity of subjects as a function of stimulus temporal frequency (*left*) and velocity (*right*). Results are presented for the three stimulus amplitudes. *Two straight lines* showing theoretical saturation, at 0.13 (*small dashes*) and 0.21°/s (*large dashes*), show the saturation levels for AP and ML, respectively. The *error bars* represent standard errors

In Fig. 3, the gain approaches zero when stimulus velocity exceeds 4°/s. As the gain corresponds to the amplitude of the postural response relative to the amplitude of the stimulus, even if the velocity of the postural response at a high stimulus velocity is substantial, its gain could be very low. For instance, consider the dash line in Fig. 3 which shows that a constant velocity of the postural response as a function of the stimulus velocity results in a rapidly decreasing gain. Thus, the gain is inadequate to illustrate variation in postural saturation (in velocity units) at high velocities. Mergner et al. (2005) show that saturation of the postural response becomes evident when examining the postural velocity of subjects. For this reason, we examined the velocity of postural response as a function of stimulus frequency and velocity for the AP (Fig. 4) and ML (Fig. 5) conditions.

One can see that the postural response to the visual stimulus can be divided into several different areas. The first comprises the postural response at low stimulus frequencies or velocities that is, before the first saturation point. The left-hand graphs in Figs. 4 and 5 show that at low frequencies, subject velocity increases as a function of frequency and amplitude. Table 1 shows that these effects are significant for both AP and ML directions except for the amplitude effect in AP that shows near significance (*p* = 0.069). Conversely, the right-hand graphs in Figs. 4 and 5 show that for low stimulus velocities, VIPR increases with velocity but very little with amplitude. This is confirmed by low F values for the amplitude effect compared to the velocity effect in Table 1. Given that velocity co-varies with amplitude and frequency, these results suggest that the postural response depends especially on stimulus velocity. This effect of velocity on postural response is only present for visual stimuli less than 0.5°/s and 0.12 Hz in the AP condition, and less than 1°/s and 0.12 Hz in the ML condition (Figs. 4, 5 left). Above these values, the postural response saturates and stimulus amplitude has no effect on mean subject velocity. This area constitutes the second area of postural response.

**Table 1:** Follow-up analyses on subsets of data (e.g. on the first/last three frequencies) were done by a linear trend analysis on the interaction term, using custom weights to extract given effects

	Visual parameters	Direction	Analysis	*p*
3 first frequencies of each curve (Figs. 4, 5 left)	A	ML	*F*(1,11) = 88.462	<0.001*
		AP	*F*(1,11) = 4.069	0.069
	F	ML	*F*(1,11) = 93.953	<0.001*
		AP	*F*(1,11) = 49.919	<0.001*
3 last frequencies of each curve (Figs. 4, 5 left)	A	ML	*F*(1,11) = 1.784	0.209
		AP	*F*(1,11) = 0.013	0.910
	F	ML	*F*(1,11) = 189.739	<0.001*
		AP	*F*(1,11) = 36.989	<0.001*
3 first velocities of each curve (Figs. 4, 5 right)	A	ML	*F*(1,11) = 26.696	<0.001*
		AP	*F*(1,11) = 6.422	0.028*
	V	ML	*F*(1,11) = 116.044	<0.001*
		AP	*F*(1,11) = 25.905	<0.001*
3 last velocities of each curve (Figs. 4, 5 right)	A	ML	*F*(1,11) = 129.573	<0.001*
		AP	*F*(1,11) = 23.194	0.001*
	V	ML	*F*(1,11) = 103.994	<0.001*
		AP	*F*(1,11) = 42.558	<0.001*

Weights for conditions corresponding to frequencies outside the range of interest were set to zero

*F* Frequency, *V* velocity, and *A* acceleration

The results show greater saturation of subject velocity in ML (0.21°/s) than in AP (0.13°/s). In order to compare these saturation levels, we did a paired samples *t* test to compare maximum above 0.25 Hz for the 12 subjects in the two directions. The difference was significant (*t* = 0.665, *df* = 12, *p* = 0.018), showing that the saturation point is significantly higher in ML than in AP.

Once this saturation is attained, increasing the stimulus frequency leads to a decrease in postural response. These decreases start between 0.25 and 0.5 Hz, regardless of the direction of the postural response. The left-hand graphs in Figs. 4 and 5 show that at high frequencies, subject velocity decreases as a function of frequency (*p* < 0.001) but with no effect of amplitude (*p* > 0.05) (see Table 1). Conversely, the right-hand graphs in Figs. 4 and 5 show that for high stimulus velocities, VIPR decreases with both velocity (*p* < 0.001) and amplitude (*p* ≤ 0.001). Results are presented in Table 1. As frequency co-varies with amplitude and velocity, these results suggest that VIPR saturation depended on the stimulus frequency and that the effect was similar for the two stimulus directions.

**Fig. 6:**
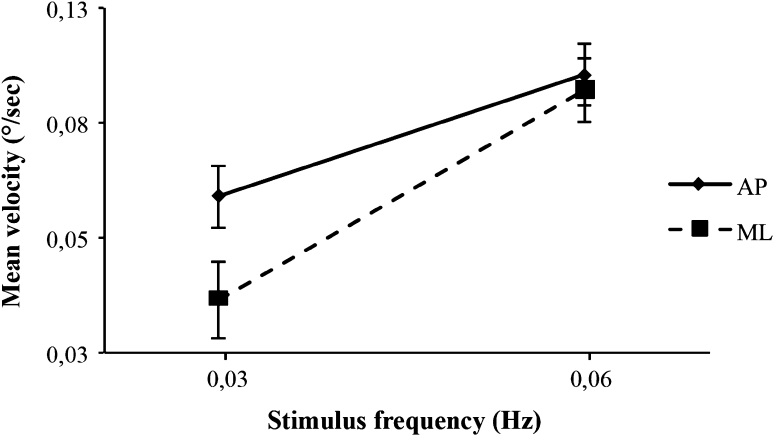
Mean subject velocity as a function of temporal frequency for the two stimulus directions. Results are presented for the two lowest temporal frequencies (0.03 and 0.06 Hz). The *error bars* represent standard errors

For the lowest frequencies (below 0.125 Hz), the visual stimulus parameters induce postural response change. One may wonder if changes in visual movement parameters influence more postural control in ML or AP direction. Figure 6 shows mean subject velocity of subjects as a function of stimulus frequency for the two directions. A repeated measures ANOVA on stimulus frequency (2) and direction (2) revealed that the frequency by direction interaction tends toward significance [*F*_(1,35)_ = 3.243, *p* = 0.080]. A more important slope in ML direction suggests that for these frequencies (0.03 and 0.06 Hz in our experiment), changes in the properties of the stimulus movement tend to affect postural response more in ML than in AP.

## Discussion

Although many studies have looked at the effects of moving visual stimuli on postural control, few have examined the physical characteristics of the dynamic scene at the origin of the visually induced postural response. For studies that have, conclusions on the respective roles of temporal frequency and velocity on the body’s oscillation have been equivocal. To address this issue, we used periodic stimuli whose movement was simple enough that we could quantify and control it in a precise way. This enabled us to independently control the temporal frequency and velocity of the movement. The results of our experiment suggest that the visually induced postural response can depend on either the frequency or the velocity of the movement, depending on the range of stimulus frequency being considered. For frequencies below 0.12 Hz, the postural response increases with the velocity of the visual stimulus. For frequencies above 0.25 Hz, the postural response depends more on temporal frequency.

In the area of low temporal frequencies, our results show that at a constant frequency, postural response increases with the amplitude of the periodic movement (Figs. 4 and 5left). At a constant velocity, there is no longer any amplitude effect (Figs. 4, 5). These data suggest, therefore, that the postural response at low frequencies is linked more to the velocity of the movement than to its frequency. Dokka et al. (2009) investigated the effect of a visual environment oscillating in the anteroposterior direction at different velocities and temporal frequencies. Their results were similar to ours, finding a significant increase in the postural response at low frequencies with an increase in stimulus velocity, while frequency had no effect. However, our results would seem to contradict the conclusions of Mergner et al. (2005). For a similar range (0.05–0.4 Hz), they reported that body oscillations depended on the frequency of the movement but not its velocity. Note, however, that we did find this effect at high frequencies (above 0.1 Hz). Furthermore, a more in-depth examination of their results shows that, for low frequencies, the postural response increased only with the amplitude of the movement, suggesting that postural changes depend more on stimulus velocity than frequency. Relevant here is another study that directly examined the effects of low stimulus velocities administered at a constant frequency (0.2 Hz) on postural response (Ravaioli et al. 2005). The results confirm that for a range of low temporal frequencies, the visual response induced by a moving stimulus depends on its velocity. In sum, all of these results suggest that at low frequencies, the postural response depends on the stimulus velocity, not its frequency.

While postural response is velocity-dependent at low frequencies, our results show that above 0.25 Hz, postural response only varies as a function of the temporal frequency of the visual stimulus. In our experiment, above this frequency, changes in amplitude, and therefore velocity, no longer affected the postural response of the subjects. These results are compatible with those of van Asten et al. (1988a, b), who observed a marked effect of frequency, but not amplitude, suggesting an effect of frequency independent of velocity, and this mainly for frequencies of 0.1 Hz and greater.

The work of Mergner et al. (2005) confirms this frequency effect, which the authors attributed “mainly to the dynamics of the postural system as a whole rather than to transfer characteristics of the visual system” (p. 545). They concluded that saturation at high frequencies may be linked to proprioceptive feedback mechanisms in the ankles, as saturation thresholds increased by a factor of 10 when this feedback was perturbed using a body sway referenced platform. In our study, postural response was measured using body sway amplitude at the stimulus frequency. The postural response depended, therefore, on the subject’s capacity to oscillate at the same frequency as the visual stimulus. Physiological studies have shown that the postural response to a visual stimulus is optimal at frequencies between about 0.2 and 0.45 Hz (Soames and Atha 1982; Gurfinkel 1974). Such a range of postural frequencies suggests that the body is not able to conserve its amplitude, and therefore its oscillation velocity, at frequencies above 0.45 Hz, a hypothesis that is supported by the subject velocity decrease we observed above 0.25 Hz. This decrease in postural response at high frequencies is associated with saturation linked to the optimal frequencies of the postural response rather than to postural saturation of a visual origin.

The results of this study show two distinct areas in postural response: one at low frequencies, where postural change is linked to the velocity of the visual stimulus, and the other at high frequencies, where the body’s response is limited by biomechanical and/or proprioceptive feedback constraints and therefore decreases as frequency increases. What, then, happens at intermediate frequencies? Our results show that the velocity of subjects reached its maximum at frequencies above 0.12 Hz. Between 0.12 and 0.25 Hz, the stimulus velocity was too high for the postural response to continue its increase, suggesting a saturation of VIPR. Maximal saturation values were higher in ML (0.21°/s) than in AP (0.13°/s) (Figs. 4, 5left). These differences in saturation confirm the results of van Asten et al., who obtained higher saturations in a tunnel swaying in the ML direction than in a tunnel making an AP translation movement (van Asten et al. 1988a, b). Mergner et al. (2005) report AP saturation of 0.10°/s, which is compatible with our result. Our results show that in ML, body oscillations saturate two times later than in AP, at around 0.21°/s. Why this difference in saturation level between the two directions? A possible explanation relates to the biomechanical constraints induced by the position of the body and feet. Anterior muscles like the tibialis anterior and posterior muscles like the soleus are very effective at stabilizing posture by maintaining the center of gravity within the base of support (Winter et al. 1998, 2001; Tia et al. 2012). By comparison, the lateral muscles are weaker but benefit from a larger base of support. When the feet are placed beside each other, the base of support becomes smaller in ML than in AP, causing greater postural excursion in the ML direction (Nichols et al. 1995). In our experiment, the subjects placed their feet together, which may explain the difference in maximal postural response between the ML and AP directions. Our results show that between 0.12 and 0.25 Hz, the difference of maximal postural response between ML and AP would depend principally on the positioning of the feet rather than on the characteristics of the visual movement.

It is primarily for visual movements at lowest frequencies (below 0.12 Hz) that the movement parameters induce postural response change. For these low frequencies, changes in the properties of the stimulus movement affected postural response more in ML than in AP (Fig. 6). This result is compatible with that of van Asten et al. (1988a, b) that used a black and white windmill or tunnel pattern subjected to different types of movement: AP translation (van Asten et al. 1988b) and ML rotation around a longitudinal axis (van Asten et al. 1988a). The results of these two studies show greater postural oscillation in ML than in AP. It would appear that postural behavior is more visually dependent in ML than in AP.

Some studies have suggested that postural response is more visually dependent for stimuli in AP than in ML (Anand et al. 2003; Winter et al. 1990; Paulus et al. 1989; Berencsi et al. 2005; Perrin et al. 1997). However, this research is referring to spatial changes as resolution, spatial frequency, brightness, distance to the viewer, size, or position on the retina and not to the dynamics of the visual environment. When it is dynamic (temporal) visual changes that are examined, the postural response is actually more affected in ML than in AP. In the experimental conditions used by van Asten et al. (1988a, b), and in our experiment, the subjects placed their feet together. This increase in visual dependence could therefore be caused, in part, by the positioning of the feet (together), which reduces the base of support in ML and therefore increases the role of visual postural control in this direction.

## Conclusion

A dynamic visual stimulus induces a compensatory postural response that enables the body to adjust to new stimulus properties. Varying the temporal frequency of a visual stimulus yields two areas of postural response. The first occurs at frequencies of less than 0.12 Hz. In this range, postural response is visually dependent and varies with stimulus velocity. The subject adjusts to their environment by increasing their postural velocity as stimulus velocity increases. This finding suggests that future research about visually induced postural response should consider using movement velocity as a stimulus parameter. In daily life, when moving the head with progressive added lenses for instance, one can see the ground moving, whereas it actually does not. Even if these optical distortions may not directly increase risks of falling, manufacturers should consider the visual distortion velocities in the designs to reduce the impact of lenses on postural control. In addition, the postural response is more pronounced in ML than in AP, contrary to what occurs when the spatial characteristics of the environment change (e.g., dimensions of the visual field). Then, between 0.12 and 0.25 Hz, postural response hits a saturation threshold: The body can no longer follow increases in the velocity of the visual scene. In this range of intermediate frequencies, maximal postural oscillation seems to depend on biomechanical constraints imposed by the positioning of the feet. The last response area is above 0.5 Hz. At these high frequencies, the body can no longer maintain the same oscillation state. This saturation may be linked to proprioceptive feedback mechanisms in the postural system.
